# Exploring the Key Amino Acid Residues Surrounding the Active Center of Lactate Dehydrogenase A for the Development of Ideal Inhibitors

**DOI:** 10.3390/molecules29092029

**Published:** 2024-04-28

**Authors:** Jie Chen, Chen Chen, Zhengfu Zhang, Fancai Zeng, Shujun Zhang

**Affiliations:** 1 Department of Biochemistry and Molecular Biology, School of Basic Medical Sciences, Southwest Medical University, Luzhou 646000, China; 20180140330134@stu.swmu.edu.cn (J.C.); 20220199120044@stu.swmu.edu.cn (C.C.); 20200140330135@stu.swmu.edu.cn (Z.Z.); 2 Key Laboratory of Southwest China Wildlife Resources Conservation, China West Normal University, Ministry of Education, Nanchong 637009, China

**Keywords:** lactate dehydrogenase A, site-directed mutagenesis, enzyme activity, molecular dynamics, inhibitors

## Abstract

Lactate dehydrogenase A (LDHA) primarily catalyzes the conversion between lactic acid and pyruvate, serving as a key enzyme in the aerobic glycolysis pathway of sugar in tumor cells. LDHA plays a crucial role in the occurrence, development, progression, invasion, metastasis, angiogenesis, and immune escape of tumors. Consequently, LDHA not only serves as a biomarker for tumor diagnosis and prognosis but also represents an ideal target for tumor therapy. Although LDHA inhibitors show great therapeutic potential, their development has proven to be challenging. In the development of LDHA inhibitors, the key active sites of LDHA are emphasized. Nevertheless, there is a relative lack of research on the amino acid residues around the active center of LDHA. Therefore, in this study, we investigated the amino acid residues around the active center of LDHA. Through structure comparison analysis, five key amino acid residues (Ala30, Met41, Lys131, Gln233, and Ala259) were identified. Subsequently, the effects of these five residues on the enzymatic properties of LDHA were investigated using site-directed mutagenesis. The results revealed that the catalytic activities of the five mutants varied to different degrees in both the reaction from lactic acid to pyruvate and pyruvate to lactic acid. Notably, the catalytic activities of LDHA^M41G^ and LDHA^K131I^ were improved, particularly in the case of LDHA^K131I^. The results of the molecular dynamics analysis of LDHA^K131I^ explained the reasons for this phenomenon. Additionally, the optimum temperature of LDHA^M41G^ and LDHA^Q233M^ increased from 35 °C to 40 °C, whereas in the reverse reaction, the optimum temperature of LDHA^M41G^ and LDHA^K131I^ decreased from 70 °C to 60 °C. These findings indicate that Ala30, Met41, Lys131, Gln233, and Ala259 exert diverse effects on the catalytic activity and optimum temperature of LHDA. Therefore, these amino acid residues, in addition to the key catalytic site of the active center, play a crucial role. Considering these residues in the design and screening of LDHA inhibitors may lead to the development of more effective inhibitors.

## 1. Introduction

Compared with normal cells, tumor cells typically exhibit changed metabolic characteristics [1,2]. Healthy cells utilize the mitochondrial tricarboxylic acid (TCA) cycle to generate energy, whereas tumor cells often depend on glycolysis for energy production, even under normal oxygen levels [3,4]. This phenomenon is termed aerobic glycolysis and is classically referred to as the “Warburg effect” [5]. Lactate dehydrogenase A (LDHA) plays a key role in this process, catalyzing the conversion of pyruvate to lactic acid in the final step of glycolysis [6].

LDHA is a protein comprising 332 amino acids, encoded by the LDHA gene located on chromosome 11p15.1, consisting of 8 exons [7]. Notably, LDHA serves as a constituent subunit (M) of lactate dehydrogenase (LDH), forming five active LDH isozymes by combining with the LDHB subunit (H) [8]. These isozymes are identified as LDH-1 (4H), LDH-2 (3H1M), LDH-3 (2H2M), LDH-4 (1H3M), and LDH-5 (4M). Notably, LDH1 and LDH5 are often referred to as LDHB and LDHA, respectively. LDHA is prevalent in hypoxic tissues, exhibiting more efficiency in catalyzing the conversion of pyruvate to lactic acid. In contrast, LDHB is more abundant in tissues with robust aerobic metabolism, prioritizing the conversion of pyruvate to acetyl-CoA and into the TCA [9]. Numerous studies have confirmed the upregulation of LDHA expression in cancer cells, whereas the expression level of LDHB remains relatively unchanged in cancer cells and normal tissues [10,11]. Current understanding validates the involvement of LDHA in various aspects of tumorigenesis, development, progression, invasion, metastasis, angiogenesis, and immune escape [10,12]. Moreover, LDHA serves as a biomarker for tumor diagnosis and prognosis, making it an attractive target for potential pharmacological interventions in cancer treatment.

Due to the significance of LDHA, extensive research has been performed on its structure and catalytic mechanism [13,14,15,16,17,18,19]. Residues 99–110 adopt a flexible “active site ring” conformation, facilitating LDHA catalysis [20]. Within this ring, Arg105 plays a crucial role in stabilizing the binding of attached pyruvate by interacting with nucleotides and substrates [21,22]. Residues 20–162 and 248–266 collectively form a large Rossmann domain. The NADH cofactor predominantly binds to four residues (Asp168, Arg171, Thr246, and catalytic His195) within the central groove of this domain [23,24,25,26]. Substrates, such as pyruvate, primarily bind to the α/β substrate binding domain, composed of residues 163–247 and 267–331, interacting with residues Arg171, Thr246, and Ala236. The active site ring, cofactor binding site, and substrate binding site collectively adopt a specific spatial conformation, actively participating in LDHA catalysis [21]. Consequently, these sites represent ideal targets for the design of inhibitors.

Notably, numerous research teams have developed LDHA inhibitors targeting the previously mentioned key catalytic sites of LDHA [5,13,27,28,29,30,31,32,33,34,35]. However, some of these inhibitors face challenges due to inadequate cellular activity. While an LDHA inhibitor exhibiting excellent cellular efficacy has been disclosed, its poor pharmacokinetics pose obstacles to in vivo evaluation [32]. The research team reported LDHA inhibitors incorporating various scaffolds [1]. Subsequently, these inhibitors were optimized, and their effects on LDHA, biochemistry, and cellular potency were assessed. Finally, a small-molecule inhibitor with cellular activity was screened [1,34,36,37,38,39]. To summarize, despite the substantial therapeutic potential of LDHA inhibition, the discovery and development of LDH inhibitors have proven to be challenging [5,33].

In the development and research of LDHA inhibitors, our primary focus revolves around the key active sites of LDHA, such as His195, Arg171, Arg109, Asp168, and Val138. However, there has been relatively little exploration of the amino acid residues surrounding the active sites of LDHA. Perhaps a more in-depth study of the roles played by these residues in LDHA catalysis could significantly contribute to the development of more optimal inhibitors.

LDHA and LDHB enzymes share approximately 75% homology in amino acid sequence, indicating their overall structural similarity, except for the residues in the substrate-binding regions [17,40]. Despite the minimal structural diversity, distinct dynamic properties characterize each form. LDHA exhibits a total turnover rate twice as high as that of LDHB, whereas LDHB shows a threefold increase in its ability to bind pyruvate [41]. These variations determine that LDHA primarily converts pyruvate into lactic acid and NADH into NAD^+^ [42]. Conversely, LDHB plays a crucial role in the kinetics of converting lactic acid to pyruvate. This study aims to identify the specific amino acid residues responsible for the observed differences by comparing the spatial structures of LDHA and LDHB. Through site-directed mutagenesis and enzymatic property analysis, we aimed to determine the contribution of these amino acids to LDHA activity and identify key residues around the active center. Therefore, in the subsequent development of inhibitors, considering these sites simultaneously may lead to the discovery of optimal inhibitors.

## 2. Results

### 2.1. Screening Differential Amino Acids by Bioinformatics

**Figure 1:**
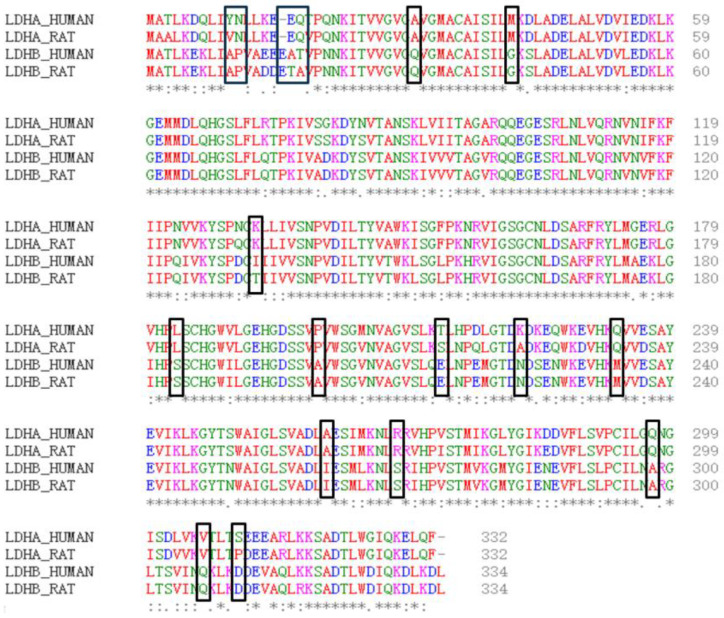
Comparison of amino acid sequences between LDHA and LDHB (human and rat). Important amino acid differences are marked with black boxes. * represents that the amino acid at this site is the same.

Firstly, the physical and chemical properties of LDHA and LDHB proteins were analyzed. The results revealed differences in certain physical and chemical properties between LDHA and LDHB proteins, such as the theoretical isoelectric point, with LDHA at 8.4 and LDHB at 5.71. Subsequently, the amino acid sequences of LDHA and LDHB proteins were compared using the EMBL-EBI online website (Figure 1), revealing 18 different amino acids between LDHA and LDHB (Table 1). Among these, 10 amino acid residues are frequently found in the active sites of catalytic reactions, including serine, tyrosine, aspartic acid, glutamic acid, and lysine.

**Figure 2:**
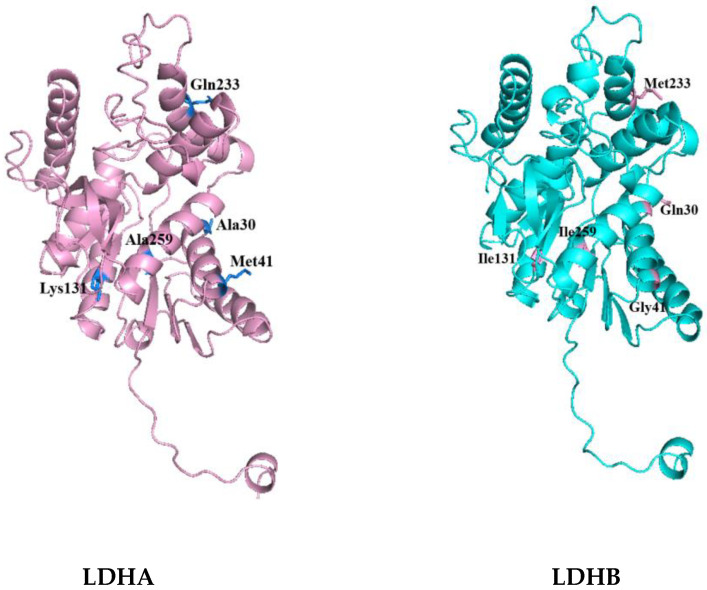
Different amino acid residues around the active sites of LDHA and LDHB.

Then, the tertiary structures of LDHA and LDHB (PDB database numbers: LDHA-6MV8; LDHB-7DBJ) were further compared and analyzed using PyMOL 2.5.8. The distribution of differential amino acids around the active sites of LDHA and LDHB proteins was analyzed using Discovery Studio 2023 software. Among the 18 differential amino acids, 5 amino acid residues were found around the active center and were included in the corresponding secondary structure. These were, for LDHA, Ala30, Met41, Lys131, Gln233, and Ala259; and, for LDHB, Gln30, Gly41, Ile131, Met233, and Ile259, as shown in Figure 2. It is worth noting that the 30th amino acid residue of the difference is also the NAD (P) binding site. The five differential amino acids in LDHA were then mutated into the corresponding amino acids in LDHB to study the contribution of these differential amino acids to the activity of LDHA.

### 2.2. Site-Directed Mutation of LDHA Gene

PCR site-directed mutagenesis was performed on the five loci of LDHA using the designed primers (Table 2). The plasmid containing the LDHA gene is preserved in our laboratory, so when the LDHA mutation sequence is amplified by PCR, the plasmid is amplified together with it. The advantage of this method is that the plasmid can be constructed simply through phosphorylation and indirectly. The expression vectors of the five mutant genes were successfully constructed through colony PCR and sequencing.

### 2.3. Expression and Purification of LDHA and Its Mutant

**Figure 3:**
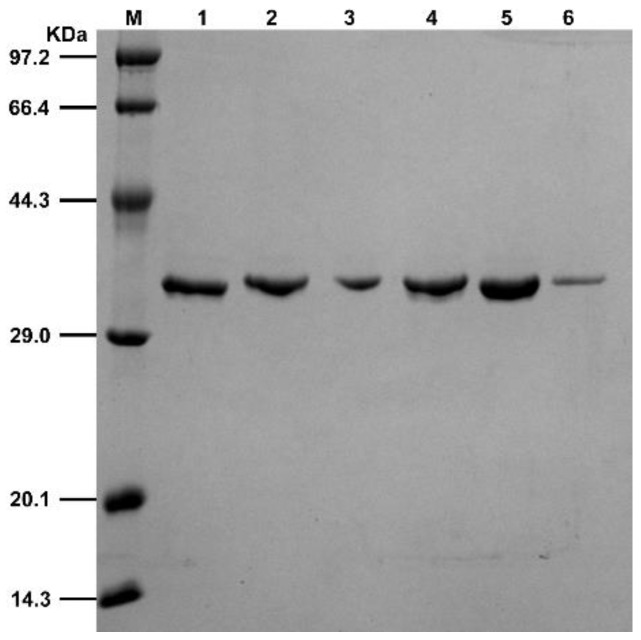
SDS-PAGE results of LDHA and its mutants. Lane M is a marker, and lanes 1–6 are LDHA, LDHA^A30Q^, LDHA^M41G^, LDHA^K131I^, LDHA^Q233M^, and LDHA^A259I^.

The aforementioned mutants were expressed and purified in *Escherichia coli* BL21 (DE3). After ultrasonic fragmentation, the supernatant was centrifuged and added to a Ni-agarose gel column. The protein was purified using different imidazole solutions (50 mM, 100 mM, and 300 mM imidazole concentrations). The purity of the protein was detected by performing SDS–PAGE. As shown in Figure 3, LDHA and its mutants A30Q, M41Q, K131I, Q233M, and A259I were successfully expressed and purified. The protein marker is the Premixed Protein Marker (Low) purchased from TaKaRa. Subsequently, the enzymatic properties of LDHA and its five mutants were analyzed.

### 2.4. Determination of Protease Activity of LDHA and Its Mutants

**Figure 4:**
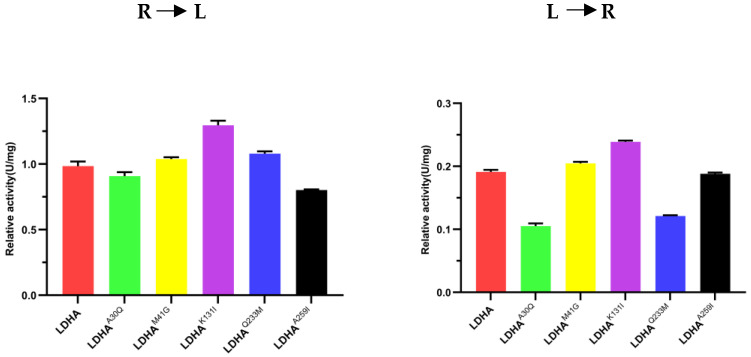
Comparison of enzyme activity between LDHA and its mutants. R to L: the reaction direction is from pyruvate to lactic acid; L to R: the reaction direction is from lactic acid to pyruvate. Each bar represents the mean standard deviation of triplicate measurements.

The concentrations of LDHA and mutant protein samples were determined using the Bradford protein concentration determination kit, and the concentration was adjusted to 0.15 mg·mL^−1^. As LDHA catalyzes a reversible reaction, the enzyme activities of LDHA and its mutant proteins were determined for both pyruvate to lactic acid and lactic acid to pyruvate, respectively. The results are shown in Figure 4.

At pH 7.5 and temperature 25 °C, the specific activity of LDHA for pyruvate reduction to lactic acid is 0.9681 U/mg, whereas the specific activity for the oxidation of lactic acid to pyruvate is 0.0982 U/mg. The specific activity of mutant LDHA^A30Q^ for pyruvate reduction to lactic acid is 0.8918 U/mg, and the specific activity for the oxidation of lactic acid to pyruvate is 0.0304 U/mg. The specific activity of the mutant LDHA^M41G^ protein for catalyzing the reduction of pyruvate to lactic acid is 1.03868 U/mg, and the specific activity for catalyzing the oxidation of lactic acid to pyruvate is 0.204567 U/mg.

The specific activity of the mutant LDHA^K131I^ for pyruvate reduction to lactic acid is 1.1904 U/mg, whereas the specific activity for the oxidation of lactic acid to pyruvate is 0.0982 U/mg. The specific activity of mutant LDHA^Q233M^ for pyruvate reduction to lactic acid is 1.0193 U/mg, and the specific activity for the oxidation of lactic acid to pyruvate is 0.0965 U/mg. The specific activity of the mutant LDHA^A259I^ for pyruvate reduction to lactic acid was 1.1949 U/mg, whereas the specific activity for the oxidation of lactic acid to pyruvate was 0.1236 U/mg.

Notably, no significant difference was found in positive reaction enzyme activity between mutant LDHA^A30Q^ and LDHA, which is 0.8918 U/mg and 0.9681 U/mg, respectively. However, the enzyme activity of LDHA in the reverse reaction is more than three times higher than that of mutant LDHA^A30Q^. Additionally, the reduction of pyruvate to lactic acid catalyzed by mutant LDHA^K131I^ was 1.2 times higher than that of LDHA, but the reverse reaction enzyme activity was the same as that of LDHA. The protease activity of mutant LDHA^A259I^ was 1.2 times higher than that of LDHA in both positive reaction and reverse reactions. These results indicate that although the amino acid residues Ala30, Lys131, and Ala259 are located around the active center of LDHA, their mutations affect the enzyme activity. This can also be demonstrated by the measured Km and Kcat (Table 3 and Table 4). Especially for LDHA^K131I^, its catalytic efficiency exhibited significant changes, whether it catalyzed the forward reaction or the reverse reaction. Hence, these amino acid sites may be potential targets for LDHA inhibitors. Therefore, when designing inhibitors for LDHA, we may be able to develop more rational inhibitors if the key sites of the active sites and the role of these sites are simultaneously considered.

### 2.5. Optimal pH Determination of LDHA and Its Mutants

**Figure 5:**
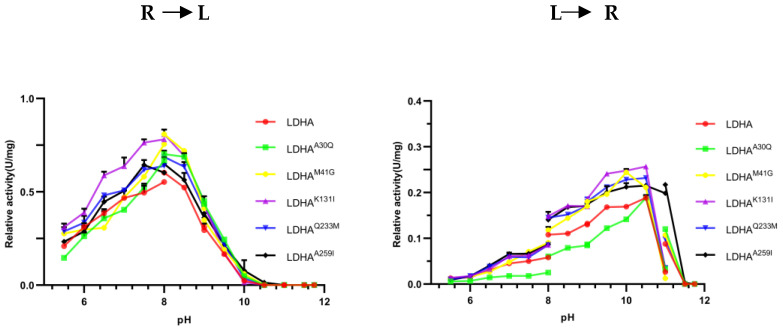
The effect of pH on the enzyme activity of LDHA and its mutants. R to L: the reaction direction is from pyruvate to lactic acid; L to R: the reaction direction is from lactic acid to pyruvate. Each bar represents the mean standard deviation of triplicate measurements.

To determine the effect of mutation sites on the optimal pH of the enzyme, the optimal pH of LDHA and its mutants LDHA^A30Q^, LDHA^K131I^, LDHA^Q233M^, and LDHA^A259I^ in the reduction of pyruvate to lactic acid and the oxidation of lactic acid to pyruvate were investigated. As shown in Figure 5, the optimum pH for the positive reaction of LDHA and mutants was 8. In the reverse reaction, except LDHA^M41G^, which was 10, the other proteins were approximately 10.3. The results indicate that the mutations of these sites had little effect on the optimal pH of LDHA, whereas the M41G mutation had a certain effect on the optimal pH of LDHA.

### 2.6. Determination of Optimal Temperature of LDHA and Its Mutants

**Figure 6:**
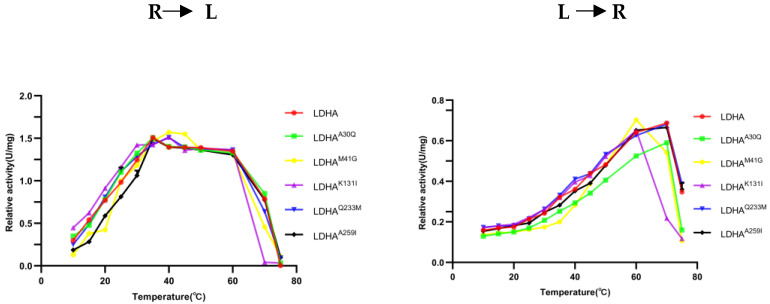
The effect of temperature on the enzyme activity of LDHA and its mutants. R to L: the reaction direction is from pyruvate to lactic acid; L to R: the reaction direction is from lactic acid to pyruvate. Each bar represents the mean standard deviation of triplicate measurements.

To determine the effect of the mutation site on the optimum temperature of the enzyme, the changes in enzyme activity of LDHA and its mutants LDHA^A30Q^, LDHA^K131I^, LDHA^Q233M^, and LDHA^A259I^ were measured at temperatures ranging from 10 °C to 75 °C. As shown in Figure 6, the optimum reaction temperature of LDHA and its mutants LDHA^A30Q^, LDHA^K131I^, LDHA^Q233M^, and LDHA^A259I^ was 35 °C. The optimum temperature of LDHA and its mutants LDHA^A30Q^, LDHA^Q233M^, and LDHA^A259I^ for the reverse reaction was 70 °C, and that of LDHA^K131I^ was 60 °C. Notably, the thermal stability of LDHA^K131I^ decreased significantly, indicating that the stability of the mutant LDHA^K131I^ protein decreased after the Lys131 mutation, and the Lys131 of LDHA may be closely related to its protein stability. Subsequent thermal stability experiments also supported this point.

### 2.7. Thermal Stability Analysis of LDHA and Its Mutants

**Figure 7:**
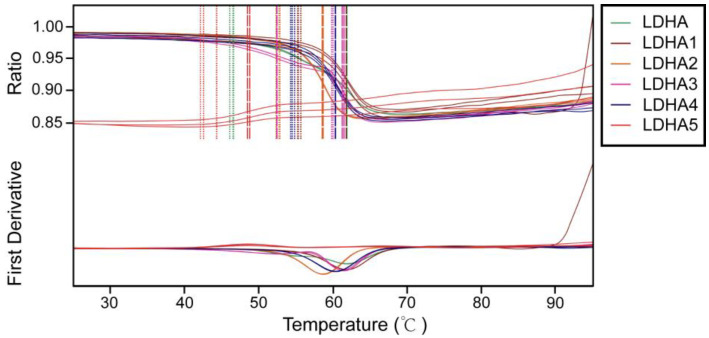
Thermal stability analysis of LDHA and its mutants. LDHA1–5 is LDHA^A30Q^, LDHA^M41G^, LDHA^K131I^, LDHA^Q233M^, and LDHA^A259I^.

To investigate the effects of mutation sites on the thermal stability of enzymes, the thermal stability of LDHA and its mutants LDHA^A30Q^, LDHA^M41G^, LDHA^K131I^, LDHA^Q233M^, and LDHA^A259I^ were determined. The results showed that compared with the wild type, mutants LDHA^A30Q^, LDHA^M41G^, LDHA^K131I^, and LDHA^Q233M^ increased the initial thermal stability temperature of the enzyme, with a minimum increase of about 6 degrees (LDHA^M41G^) and a maximum increase of about 13 degrees (LDHA^K131I^) (Figure 7 and Table 5). However, mutant LDHA^A259I^ weakened the thermal stability of the protein. These results indicate that these five sites play important roles in maintaining the thermal stability of LDHA.

### 2.8. Molecular Docking

**Figure 8:**
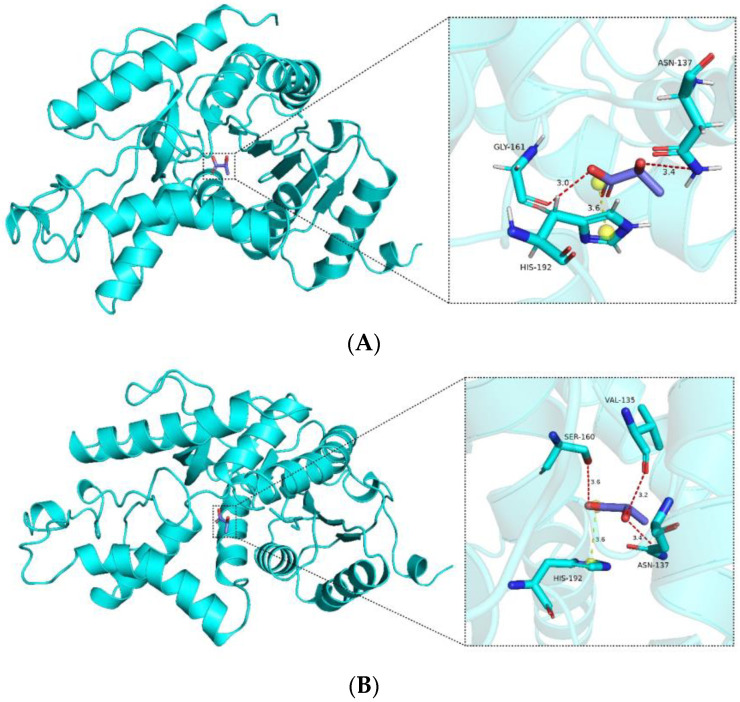
Analysis of the interaction between wild-type LDHA (**A**) and mutant K131I (**B**) with substrates.

From the docking analysis, it is evident that interactions occur between the compound and the protein. According to the Vina binding energy prediction algorithm based on the AMBER force field, the predicted binding energies between the ligand and both the wild-type LDHA and mutant K131I are −4.1 kcal/mol. Using the PLIP server, interactions in the protein–ligand complex were analyzed, and the results were visualized using PyMOL. As shown in Figure 8, the protein forms a number of hydrogen bond interactions (red) and electrostatic interactions (yellow) with the carboxyl group of the compound. Specific interacting residues are labeled in Figure 8, with the lengths of the interaction bonds marked in angstroms next to the dashed lines. It can be observed that the mutant K131I forms a greater number of hydrogen bonds with the substrate (Figure 8B). However, no difference in binding energy is observed between the two complexes. Further detailed analysis will be required in conjunction with subsequent molecular dynamics simulations. Additionally, it is noted that the binding cavities of both proteins with the substrate are located near the 131st amino acid residue. In the mutant K131I complex, the small molecule is relatively closer to the 131st amino acid residue, suggesting the possibility of enhanced catalytic activity following the mutation.

### 2.9. Molecule Dynamics

**Figure 9:**
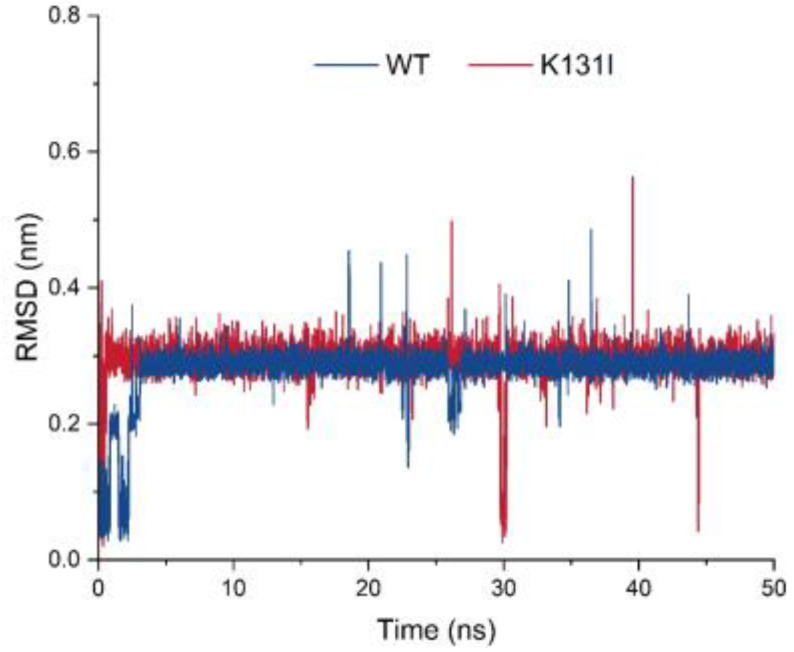
Changes in the root mean square displacement (RMSD) of LDHA (WT) and the mutant (K131I).

Before conducting molecular dynamics simulation, we employed CASTp to investigate the characteristics of mutation sites and discovered that the mutations had a certain impact on the cavities of the protein (Table S1). Subsequently, molecular dynamics analysis was performed on the mutant K131I, which exhibited a significant increase in activity. The RMSD curve displays the root mean square displacement of the small molecule relative to the protein during the simulation (Figure 9). From the analysis of the graph, it can be observed that throughout the entire simulation process, the RMSD curve of the small molecule relative to the WT protein undergoes some fluctuations in the initial 5 ns of simulation before stabilizing around 0.3 nm and oscillating around this value until the end of the simulation. There is a decrease near 25 ns followed by an upward trend to around 0.2 nm before returning to 0.3 nm. On the other hand, the RMSD curve of the small molecule relative to the K131I protein rapidly increases to 0.3 nm within the first 1 ns of simulation and then remains stable around 0.3 nm until the end of the simulation.

Analysis of the trajectory of the small molecule–protein complex movement reveals that when the small molecule binds to the WT protein, there is noticeable positional movement relative to the protein in the first 5 ns of simulation before stabilizing. During this period, the small molecule maintains changes in its conformation within the binding cavity of the protein, with a brief detachment from the binding cavity near 25 ns followed by gradual reattachment. In contrast, when the small molecule binds to the K131I protein, it rapidly enters the binding cavity within the initial 1 ns of simulation and maintains stable binding within the cavity throughout the simulation. It is speculated that the change in hydrophobic surface area within the cavity due to the K131I mutation allows the small molecule to enter the protein’s binding cavity faster compared to the WT protein and subsequently stabilize its conformation within the cavity during the simulation. This forms certain interactions, resulting in a more stable complex compared to WT, which can be further analyzed through MMGBSA energy analysis for comparison.

**Figure 10:**
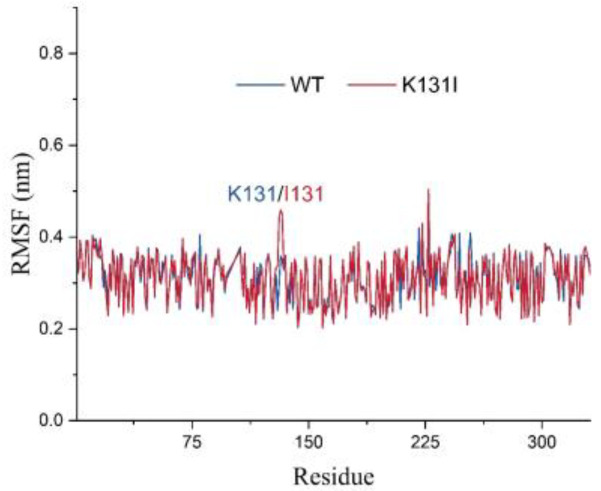
The RMSF analysis of LDHA (WT) and the mutant (K131I).

The RMSF analysis illustrates the adaptability of each residue in the protein, and in this study, it was utilized to analyze the flexibility and degree of motion of amino acid residues throughout the entire simulation process (Figure 10). From the graph, it can be observed that the overall RMSF of the protein remains relatively low throughout the simulation. The higher RMSF values at the N- and C-termini of the protein are likely due to these regions being located at the ends of the protein, experiencing fewer constraints from the rest of the protein structure, thus exhibiting greater flexibility, which affects their stability. Additionally, the higher RMSF values in other parts of the protein may be attributed to perturbations caused by the binding of the small molecule, or due to inherent flexibility of peptide segments experiencing disturbances during the simulation.

It is worth noting that after mutating K131 to I131, there is an increase in RMSF values for residue 131 and nearby peptide segments. Combined with the RMSD analysis mentioned earlier, it can be speculated that this may be because the mutated I131 maintains certain interactions with the small molecule throughout the simulation while the small molecule adjusts its structure within the cavity. Consequently, under the pulling effect of the small molecule, the flexibility of residue 131 and nearby peptide segments is influenced, resulting in increased RMSF values. Overall, the relatively low RMSF values of the protein suggest minimal vibration and stable oscillation throughout the simulation, indicating reliable sampling and analysis of the protein in the solvent environment.

**Figure 11:**
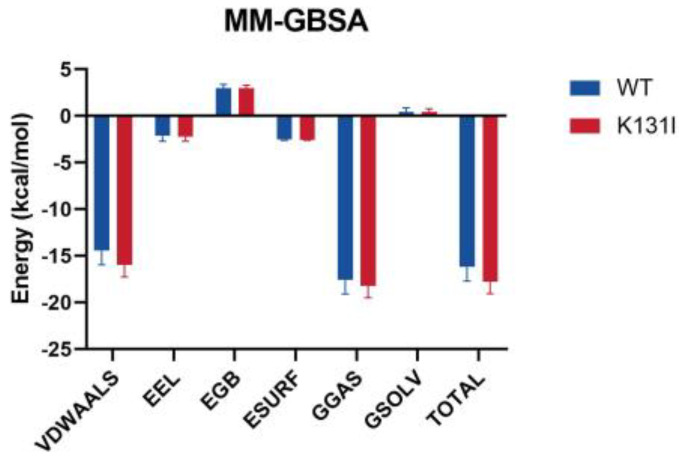
The binding free energy (MM/GBSA) analysis of LDHA (WT) and the mutant (K131I). Note: ΔE-vdw represents van der Waals energy, ΔE-elec represents electrostatic energy, ΔE-gas represents gas-phase free energy, ΔE-gas = ΔE-vdw + ΔE-elec; ΔE-surf represents nonpolar solvent energy, ΔE-GB represents polar solvent energy, ΔE-solv represents solvent energy, ΔE-solv = ΔE-GB + ΔE-surf; ΔE-Bind represents overall binding free energy, ΔE-Bind = ΔE-gas + ΔE-solv.

After performing periodic removal on the simulation trajectory, the binding free energy (MM/GBSA) analysis of the small molecule–protein complex was conducted, and the energy was decomposed into individual terms (Figure 11). The results indicate that the binding energy between the small molecule and the two proteins is primarily dominated by van der Waals interactions, with electrostatic contributions being relatively minor. It is noted that in the residue energy decomposition, a significant number of interacting residues between the protein and the small molecule are hydrophobic residues. Furthermore, as mentioned earlier in the RMSD analysis, the small molecule maintains conformational changes within the protein’s binding cavity, suggesting that van der Waals interactions play a major role in the interaction between the small molecule and the protein.

The main differences in the MMGBSA energy terms between WT and K131I proteins are also primarily reflected in the van der Waals energy term. It is speculated that mutating the hydrophilic amino acid K131 to the hydrophobic amino acid I131 further enhances the hydrophobicity of the cavity, making it more favorable for small molecule binding. Additionally, solvent solvation energy hardly contributes to the binding in this simulation, further confirming van der Waals energy as the main energy contribution.

The overall binding energy is −16.17 kcal/mol for WT and −17.79 kcal/mol for K131I, slightly higher than that of the WT protein, indicating a greater binding tendency between the small molecule and the protein. From an energetic perspective, this further elucidates the stability of the small molecule binding to the K131I protein.

## 3. Discussion

In this study, comparisons of the tertiary structures of LDHA and LDHB and the distribution of differential amino acids around the active sites of LDHA and LDHB proteins revealed five distinct amino acid residues around the active center, which are incorporated into their respective secondary structures: LDHA (Ala30, Met41, Lys131, Gln233, and Ala259) and LDHB (Gln30, Gly41, Ile131, Met233, and Ile259). To understand the effect of these differential amino acids on LDHA activity, five mutations (LDHA^A30Q^, LDHA^M41G^, LDHA^K131I^, LDHA^Q233M^, and LDHA^A259I^) were introduced through site-directed mutagenesis. The results showed varying degrees of change in catalytic activities for the mutants, with improvements observed in LDHA^M41G^ and LDHA^K131I^, especially in the case of LDHA^K131I^. Through molecular dynamics simulation analysis of LDHA^K131I^, it is speculated that the reason for this phenomenon may be the further enhancement of the hydrophobicity of the cavity after mutating the hydrophilic amino acid K131 to the hydrophobic amino acid I131, which subsequently favors the binding of small molecules. The overall binding energy is −16.17 kcal/mol for the LDHA and −17.79 kcal/mol for the LDHA^K131I^, indicating a greater tendency for small molecules to bind to the protein. From the perspective of energy, this further elucidates the stability of the binding between small molecules and LDHA^K131I^. Thus, LDHA^K131I^ may be a favorable choice for improving LDHA activity. We also determined the effects of these five mutations on the optimum temperature of LDHA. While LDHA^A30Q^, LDHA^K131I^, and LDHA^A259I^ exhibited no change in the optimum temperature for the positive reaction (35 °C, similar to wild-type LDHA), LDHA^M41G^ and LDHA^Q233M^ showed an increase to 40 °C. Conversely, in the reverse reaction, the optimum temperature of LDHA^M41G^ and LDHA^K131I^ decreased to 60 °C (70 °C for other mutants and wild-type LDHA). Altogether, these results indicate that the loci M41G, K131I, and Q233M exert different effects on the optimum temperature of LDHA.

Subsequently, the effect of the mutation site on the optimal pH of the enzyme was investigated. The results showed that compared with the wild-type LDHA, no significant change was found in the optimal pH of the positive and inverse response of the mutant (the optimum pH of the positive response was 8, and the optimum pH of the reverse reaction was 10). Lastly, the effect of the mutation site on the thermal stability of the enzyme was investigated. The results showed that these five sites play important roles in maintaining the thermal stability of LDHA. In fact, we are currently screening inhibitors targeting these sites. The integration of structure-based virtual screening with biological experimental validation has proven to be an effective strategy in the search for relevant drugs. Additionally, the employment of bioinformatics techniques such as molecular docking, molecular dynamics simulation, and free energy analysis enhances the reliability of virtually screened drugs and facilitates the elucidation of the interaction mechanisms between targets and drugs. Presently, we have completed the virtual screening targeting these five residue sites as well as known critical active sites, resulting in a list of 100 potential inhibitors ranked by their scores. Subsequent steps involve the design of biological experiments for validation and the analysis of their mechanistic actions.

## 4. Materials and Methods

### 4.1. Bioinformatics Analysis

The amino acid sequences of human LDHA and LDHB proteins were searched for and downloaded from the National Bioinformatics Center website. The Expasy online platform, specifically the Expasy-ProtParam tool (https://web.expasy.org/protparam/. accessed date: 1 February 2022), was utilized to analyze the physical and chemical properties of LDHA and LDHB, such as the number of positive and negative charges and hydrophobicity. The amino acid sequences of the proteins were compared using the EMBL-EBI online platform to identify differences. The crystal structures of LDHA and LDHB were then downloaded from the Protein Data Bank (PDB) database. The PyMOL software was employed to compare the secondary structures and overlay the tertiary structures. Finally, the amino acid residues around the active site were predicted using Discovery Studio software.

### 4.2. Cloning and Site-Directed Mutagenesis of LDHA

The plasmid containing the LDHA gene was stored in our laboratory. Primers were designed based on the mutation site, and PCR site-directed mutagenesis was performed using a high-fidelity enzyme kit (Fermentas) (Table 2). The amplification reaction conditions were as follows: 98 °C for 1 min, 98 °C for 10 min, 50 °C for 45 s, 72 °C for 3 min, and 72 °C for 4 °C. The PCR products were verified by agarose gel electrophoresis. Subsequently, the LDHA mutant gene was constructed into the expression vector pET28a through PCR product recovery, phosphorylation, and ligation steps. Finally, LDHA and its mutants were expressed as fusion proteins with the His-tag carried by the vector pET28a. The His-tag was used for the purification of the target proteins. The plasmid was then introduced into *E. coli* DH5α competent cells using the heat shock method. After heat shock treatment, 0.6 mL LB medium was added, and the cells were cultured in a 180 rpm shaker at 37 °C for 60 min. The transformed *E. coli* was evenly spread onto agarose plates containing kanamycin and incubated overnight at 37 °C. Positive clones were screened through colony PCR. The correct monoclonal colonies identified by PCR were expanded and further confirmed by sequencing. The validated strain was preserved for future use.

### 4.3. Prokaryotic Expression of 3LDHA and Its Mutants

The successfully constructed expression vector was introduced into the recipient strain BL21 (DE3) using the heat shock method. Kanamycin resistance of the expression vector was employed to screen for positive bacteria. The selected positive bacteria were inoculated into 5 mL of liquid LB medium, and kanamycin was added to the medium at a final concentration of 50 µg/mL. The culture was incubated overnight at 37 °C under 220 rpm conditions. Subsequently, 1 mL of the overnight culture was transferred to 1 L of LB medium containing 50 µg/mL kanamycin. When the culture reached an OD_600nm_ 0.8 at 37 °C, IPTG was added to induce the expression of the nucleocapsid protein, with a final concentration of 0.2 mM. The induction was carried out at 20 °C under 220 rpm for 12 h. Following induction, the bacteria were harvested by centrifugation at 5000 rpm for 10 min.

### 4.4. Protein Purification

The collected bacteria were re-suspended, and the suspension buffer was divided into 50 mM NaH_2_PO_4_, 300 mM NaCl, 10 mM Imidazole, 10% glycerol, 1 mM PMSF, DNase I, and RNase A, at pH 8.0. Subsequently, ultrasonic treatment was applied for cell lysis. The lysate was centrifuged at 12,000× *g* rpm for 30 min at 4 °C, and the resulting clarified supernatant was filtered through a 0.45 µM filter before purification using a Ni-agarose gel 6FF column. Impurity proteins were washed with a buffer containing 50 mM and 100 mM Imidazole, whereas the target protein was eluted using a buffer containing 300 mM Imidazole. The corresponding eluate was collected for SDS-PAGE identification.

### 4.5. Enzyme Activity Analysis

The activity of LDHA and its mutant proteins, LDHA^A30Q^, LDHA^M41G^, LDHA^K131I^, LDHA^Q233M^, and LDHA^A259I^, was determined using a UV-vis spectrophotometer. For the reaction mixture involving the reduction of pyruvate to lactic acid, 10 µL of enzymes (100 mmol/L Tris-HCl, pH = 7.5), 0.15 mmol/L NADH, 1.0 mmol/L pyruvate, and 0.15 mg/mL were combined, resulting in a final reaction volume of 1.5 mL. On the other hand, the oxidation mixture for lactic acid to pyruvate included 10 µL of enzymes (100 mmol/L Tris-HCl, pH = 7.5), 20 mM sodium lactate, 1 mM NAD+, and 0.15 mg/mL, with a final reaction volume of 1.5 mL. The reaction progress was monitored by tracking the change in absorbance at 340 nm at 25 °C. Each measurement was repeated three times, and the calculated specific activity was expressed as U/mg protein.

### 4.6. Km and Kcat Measurements

Under the conditions of pH 7.5 and 25 °C, the Km values of LDHA and mutant proteins were measured for four substrates: pyruvate, NADH, lactic acid, and NAD^+^, with final substrate concentrations of 0.1 to 1.2 mmol·L^−1^ for pyruvate, 0.04 to 0.8 mmol·L^−1^ for NADH, 2 to 64 mmol·L^−1^ for lactic acid, and 0.1 to 3.2 mmol·L^−1^ for NAD^+^. The Km and Kcat values were calculated using the software GraphPad Prism 9.5 through nonlinear regression fitting of the Michaelis–Menten equation.

### 4.7. Optimum Temperature Measurement

The positive reaction mixture, containing 100 mmol/L Tris-HCl (pH = 7.5), 0.15 mmol/L NADH, and 1.0 mmol/L pyruvate, was incubated in the temperature range of 10 °C to 75 °C, with the enzyme incubated separately at the corresponding temperature. Subsequently, the mixture at different temperatures was combined with the enzyme, and the enzyme amount was 0.15 mg/mL LDHA (10 µL). The absorbance at OD_340_ was then measured after a 1 min reaction at the corresponding temperature. The optimum temperature of the reverse reaction (conversion of lactic acid to pyruvate) was determined using the same method. The reverse reaction mixture consisted of 100 mmol/L Tris-HCl (pH = 7.5), 20 mM sodium lactate, and 1 mM NAD^+^. Each measurement was repeated three times, and the calculated specific activity was expressed as U/mg protein.

### 4.8. Optimal pH Measurement

Four different pH buffering systems were used to determine the optimal pH of purified LDHA and its mutants in the direction of reduction of pyruvate to lactic acid and oxidation of lactic acid to pyruvate, respectively. Of note, 100 mM PBS (pH 5.5, 6.0, 6.5, 7.0, 7.5, and 8.0); 100 mM Tris-HCl (pH 8.0, 8.5, and 9.0); 100 mM Glycine—NaOH (pH 9.0, 9.5, 10.0, 10.5, and 11.0); 100 mM Na_2_HPO_4_—NaOH (pH 11.0, 11.5, and 11.75) were used. Subsequently, the enzyme activity was determined at 25 °C.

### 4.9. Thermal Stability Analysis of LDHA and Its Mutants

The thermal stability of LDHA and its mutants was determined by the protein stability analysis system PrometheusNT.48. Tyrosine and tryptophan of the samples were detected by 330 nm and 350 nm autofluorescence. The sample in the capillary tube was measured within 3 s, and the unfolding transition was detected. The temperature was raised from 15 °C to 110 °C.

### 4.10. Analysis of Mutation Site Characteristics

The protein was mutated at the mutation site using Discovery Studio. Subsequently, the results were prepared using PyMoL and uploaded to the CASTp 3.0 server (http://sts.bioe.uic.edu/castp/ (accessed on 1 March 2022)) for hydrophobic area calculation and cavity detection.

### 4.11. Molecular Docking

The protein crystal structure was obtained from the RCSB PDB database (PDB ID: 6MV8). The obtained 3D protein structure was prepared, including hydrogenation, using PyMoL 2.5.8. The compound selected was lactate, an electrostatic substrate of lactate dehydrogenase A (LDHA), obtained from the PubChem database (https://pubchem.ncbi.nlm.nih.gov/ (accessed on 16 April 2024)). Docking was performed using the CB-DOCK2 online server (https://cadd.labshare.cn/cb-dock2/php/blinddock.php (accessed on 16 April 2024)). CB-DOCK2 employs artificial neural networks for cavity detection and utilizes AutoDock Vina for docking. Docked complexes were selected based on binding energy, followed by force analysis and docking position evaluation for subsequent validation of binding stability. Analysis of interactions between ligands and receptors was conducted using the PLIP online server (https://plip-tool.biotec.tu-dresden.de/plip-web (accessed on 16 April 2024)), and the 3D conformations of ligand–receptor complexes were visualized using PyMoL 2.5.8 software.

### 4.12. Molecule Dynamics

Based on the docked small molecule–protein complexes obtained, initial structures were subjected to all-atom molecular dynamics simulations using the Gromacs 2020.6 software. The small molecules were described using the GAFF force field, while the proteins were described using the AMBER protein force field. The pdb2gmx subroutine was employed to add hydrogen atoms to the system. A truncated cubic TIP3P solvent box was added at a distance of 10 Å from the system, and Na^+^/Cl^−^ ions were added to balance the system’s charge. Finally, topology and parameter files were generated for simulation. Molecular dynamics simulations were conducted using the Gromacs 2020.6 software for a duration of 50 ns. Prior to simulation, the system was energy minimized using the mdrun command and the steepest descent method (canonical ensemble), with a starting step size of 0.01 nm and a maximum force tolerance of 1000 kJ/mol·nm. After energy minimization, the system underwent a 100 ps NVT (isothermal–isochoric) ensemble simulation under fixed volume and constant heating rate, gradually raising the temperature from 0 K to 310.15 K to ensure further uniform distribution of solvent molecules in the solvent box. Subsequently, a 100 ps NPT (isothermal–isobaric) ensemble simulation was performed using the Berendsen barostat to equilibrate the pressure of the solvent and complex system to 1 bar. During MD simulation, all hydrogen bonds were constrained using the LINCS algorithm with a time step of 2 fs. Electrostatic interactions were calculated using the particle-mesh Ewald (PME) method with a cutoff of 1.2 nm. Non-bonded interactions were truncated at 10 Å with updates every 10 steps. The resulting trajectories were subjected to periodic removal, followed by subsequent analyses such as RMSD, RMSF, and MMGBSA.

### 4.13. MMGBSA Binding Free Energy Calculation

The binding free energy between the small molecule and the protein was calculated using the MM/GBSA method. The specific formula is as follows:

(1) $$\begin{array}{c} {\Delta G}_{bind}={\Delta G}_{complex}-\left({\Delta G}_{receptor}+{\Delta G}_{ligand}\right)\\ ={\Delta E}_{internal}+{\Delta E}_{VDW}+{\Delta E}_{elec}{+\Delta G}_{GB}+{\Delta G}_{SA} \end{array}$$

In Equation (1), ΔE_internal represents internal energy, ΔE_VDW represents van der Waals interactions, and ΔE_elec represents electrostatic interactions. The internal energy includes bond energy (E_bond), angle energy (E_angle), and torsional energy (E_torsion). ΔG_GB and ΔG_GA collectively represent solvation-free energy, where ΔG_GB is the polar solvation-free energy, and ΔG_SA is the nonpolar solvation-free energy. For ΔG_GB, this study employed the GB model developed by Nguyen et al. with igb = 2. The nonpolar solvation free energy (ΔG_SA) is calculated based on the product of surface tension (γ) and the solvent-accessible surface area (SA), where ΔG_SA = 0.0072 × ΔSASA. Entropy change, due to high computational costs and low precision, was neglected in this study.

## 5. Conclusions

LDHA is involved in various stages of cancer, including tumorigenesis, development, progression, invasion, metastasis, angiogenesis, and immune escape. It serves as a crucial biomarker for tumor diagnosis and prognosis, making it an attractive target for potential pharmacological interventions in cancer treatment. In the development and research of LDHA inhibitors, the researchers mainly focus on the key active sites of LDHA, namely His195, Arg171, Arg109, Asp168, and Val138. However, the amino acid residues around the active sites of LDHA have not been well studied. Perhaps more effective inhibitor targets can be identified from these amino acids. In this study, we investigated the amino acid residues surrounding the active center of LDHA. Through structural comparison and analysis, five key amino acid residues were identified, and their roles in the physicochemical properties and catalytic mechanisms of lactate dehydrogenase were studied. The findings underscore the significance of amino acid residues Ala30, Met41, Lys131, Gln233, and Ala259 in influencing the catalytic activity, optimum temperature, and thermal stability of LDHA. These residues, in addition to the key catalytic site within the active center, play a crucial role. In the process of designing and screening inhibitors for LDHA, considering the effect of these residues could lead to the development of more effective inhibitors.

## Figures and Tables

**Table 1:** The differences in amino acids between LDHA and LDHB.

Residue Site	10	11	15	16	17	30	41	131	183	199	213	222	233	259	267	296	305	309
LDHA	Y	N	-	E	Q	A	M	K	L	P	T	K	Q	A	R	Q	V	S
LDHB	A	P	E	A	T	Q	G	I	S	A	E	N	M	I	S	A	Q	D

**Table 2:** Primers are used to amplify LDHA and its mutants.

Primer Name	Primer Sequence (5′ to 3′)
LDHA—forward primer	ATGGCAACTCTAAAGGATCAGCTGA
LDHA—reverse primer	AAATTGCAGCTCCTTTTGGATCCCC
LDHA^A30Q^—forward primer	GTTGGCATGGCCTGTGCCATCAGTA
LDHA^A30Q^—reverse primer	TTGACCAACCCCAACAACTGTAATC
LDHA^M41G^—forward primer	TTGGCAGATGAACTTGCTCTTGTTG
LDHA^M41G^—reverse primer	GTCCTTTCCTAAGATACTGATGGCA
LDHA^K131I^—forward primer	TTGCTTATTGTTTCAAATCCAGTGG
LDHA^K131I^—reverse primer	GATGCAGTTCGGGCTGTATTTTACA
LDHA^Q233M^—forward primer	ATGGTGGTTGAGAGTGCTTATGA
LDHA^Q233M^—reverse primer	CTTGTGAACCTCTTTCCACTGT
LDHA^A259I^—forward primer	ATTGAGAGTATAATGAAGAATCTTA
LDHA^A259I^—reverse primer	CAAATCTGCTACAGAGAGTCCAATA

**Table 3:** Determination of Km and Kcat for the reaction catalyzed by LDHA and its mutants to convert pyruvate into lactate acid.

Enzyme	LDHA		LDHA^A30Q^		LDHA^M41G^		LDHA^K131I^		LDHA^Q233M^		LDHA^A259I^	
Substrate	Pyruvate ^1^	NADH ^2^	Pyruvate	NADH	Pyruvate	NADH	Pyruvate	NADH	Pyruvate	NADH	Pyruvate	NADH
Km (mmol/L)	0.4788	0.1055	0.6753	0.1114	0.4615	0.1046	0.4592	0.08781	0.4660	0.09130	1.036	0.1768
Kcat (1/s)	11.32	7.507	10.87	7.273	11.37	7.547	12.61	15.41	11.81	10.14	12.24	7.852
Kcat/Km (L/mmol∙s)	23.64	71.16	16.10	65.29	24.64	72.15	27.46	175.49	25.34	111.06	11.81	44.41

^1^ Km and Kcat were measured when the concentration of pyruvate was a variable, and ^2^ Km and Kcat were measured when the concentration of NADH was a variable.

**Table 4:** Determination of Km and Kcat for the reaction catalyzed by LDHA and its mutants to convert lactate acid into pyruvate.

Enzyme	LDHA		LDHA^A30Q^		LDHA^M41G^		LDHA^K131I^		LDHA^Q233M^		LDHA^A259I^	
Substrate	Lactic Acid ^3^	NAD^+ 4^	Lactic Acid	NAD^+^	Lactic Acid	NAD^+^	Lactic Acid	NAD^+^	Lactic Acid	NAD^+^	Lactic Acid	NAD^+^
Km (mmol/L)	17.66	0.8878	32.83	2.509	16.68	0.8381	11.10	0.6038	21.90	1.529	18.35	0.8913
Kcat (1/s)	3.180	3.062	1.076	0.366	3.088	2.971	3.199	3.029	3.004	0.334	3.264	3.015
Kcat/Km (L/mmol∙s)	0.18	3.45	0.03	0.15	0.19	3.54	0.29	5.017	0.14	0.22	0.18	3.38

^3^ Km and Kcat were measured when the concentration of lactic acid was a variable; ^4^ Km and Kcat were measured when the concentration of NAD^+^ was a variable.

**Table 5:** Unfolding temperature of LDHA and its mutants.

Sample ID	Onset #1 for Ratio	Inflection Point #1 for Ratio
LDHA	46.4 °C	61.8 °C
LDHA1(A30Q)	55.6 °C	61.8 °C
LDHA2(M41G)	52.6 °C	58.6 °C
LDHA3(K131I)	59.9 °C	61.4 °C
LDHA4(Q233M)	54.5 °C	60.3 °C
LDHA5(A259I)	43.0 °C	48.6 °C

## Data Availability

Data are contained within the article.

## Supplementary Materials

The following supporting information can be downloaded at: https://www.mdpi.com/article/10.3390/molecules29092029/s1, Table S1: The results of the structural characteristics of LDHA and its mutants using CASTp.
